# Pseudovascular squamous cell carcinoma of the buccal mucosa—a rare case report and review of literature

**DOI:** 10.3332/ecancer.2024.1802

**Published:** 2024-11-18

**Authors:** Rituparna Dhekial, Rukmini Bezbaruah, Arpan Choudhury, Anupam Das, Sakshi Gupta

**Affiliations:** 1 Department of Oncopathology, Dr. B. Borooah Cancer Institute, Guwahati 781016, Assam, India; 2 Department of Surgical Oncology, Dr. B. Borooah Cancer Institute, Guwahati 781016, Assam, India; 3 Department of Head and Neck Surgery, Dr. B. Borooah Cancer Institute, Guwahati 781016, Assam, India

**Keywords:** Pseudovascular squamous cell carcinoma, acantholytic squamous cell carcinoma, histopathology case report, oral cavity

## Abstract

Squamous cell carcinoma is the most common malignancy of the head and neck. Pseudovascular squamous cell carcinoma (PSCC) is a rare variant that occurs commonly in the skin of the head and neck. However, oral cavity involvement is extremely rare, with only a few cases reported to date. This variant is associated with an adverse prognosis. Morphologically, the tumour shows marked acantholysis and anastomosing channels mimicking vascular neoplasms. This rare variant poses a diagnostic challenge for pathologists and the accurate diagnosis can be made with the help of immunohistochemical (IHC) studies. A 72-year-old male patient was referred to our hospital with a fast-growing left-sided oral ulcer for more than 1 month. Wide local excision with segmental mandibulectomy was performed and the specimen was sent to our department. On gross examination, a large, soft, lobulated mass was identified in the lower alveolus and the buccal mucosa. On microscopic examination, the tumour was composed of malignant epithelial cells arranged in nests, trabeculae and irregular anastomosing channels. Marked acantholysis and pseudo vascular spaces lined by atypical cells were seen. IHC examination revealed tumour cells positive for CK, P40, P63 and Vimentin and negative for CD31, CD34, S100 and HMB45. The pathological diagnosis was confirmed as PSCC. A short history of the case and a review of literature is discussed here.

## Introduction

Pseudovascular squamous cell carcinoma (PSCC) is a rare but a well-defined histologic variant of squamous cell carcinoma (SCC), characterised by formation of anastomosing spaces and channels. These features mimic angiosarcoma, so the other name for this variant is angiosarcoma like SCC or pseudoangiosarcomatous carcinoma. PSCC is a subtype of acantholytic SCC (ASCC). ASCC carries a poor prognosis and more chances of recurrence and metastasis than SCC of the skin as it commonly occurs in the sun exposed areas of the head and neck region. However, ASCC is an extremely rare disease in the oral cavity with only a few cases reported in the literature. Here, we describe a rare case of PSCC of the buccal mucosa, highlighting the clinical manifestations, the immunohistochemical (IHC) profile leading to its diagnosis and also the literature review of the previously published related cases.

## Case report

A 72-year-old male was referred to our hospital with a fast-growing left-sided oral ulcer for more than 1 month. He presented with mild pain during swallowing and numbness. On local examination, there was a 6 × 4 cm lesion on the left buccal mucosa and left lower alveolus. Computed tomography scan revealed a large heterogenous lesion in the left side of the oral cavity with encroachment of the lower gingivobuccal sulcus and angle of mouth. There was an enlargement of the left level IB node. He had a past history of carcinoma of the right buccal mucosa, for which he had undergone wide local excision and marginal mandibulectomy at our hospital many years back. There was another past history of a thigh lesion, histopathology of which was suggestive of Pleomorphic spindle cell sarcoma and IHC panels consisting of CK, Vimentin, CD31, Desmin, Myogenin, S100, HMB 45 and CD34 was advised, for which he received adjuvant radiotherapy, but proper documentation was not available and the patient was lost to follow up. The patient had undergone two biopsies of the said lesion of the left buccal mucosa, first was reported as no opinion possible and second which was reported as suspicious of pyogenic granuloma. But based on clinical suspicion after explaining all the risks and benefits, he underwent wide local excision of the mass of the left buccal mucosa and segmental mandibulectomy and left Modified radical neck dissection was performed. The defect was reconstructed using a Pectoralis Major Myocutaneous flap. The procedure and postoperative recovery were uneventful. The specimen was sent to our histopathology laboratory under Department of Oncopathology.

**Figure 1.**
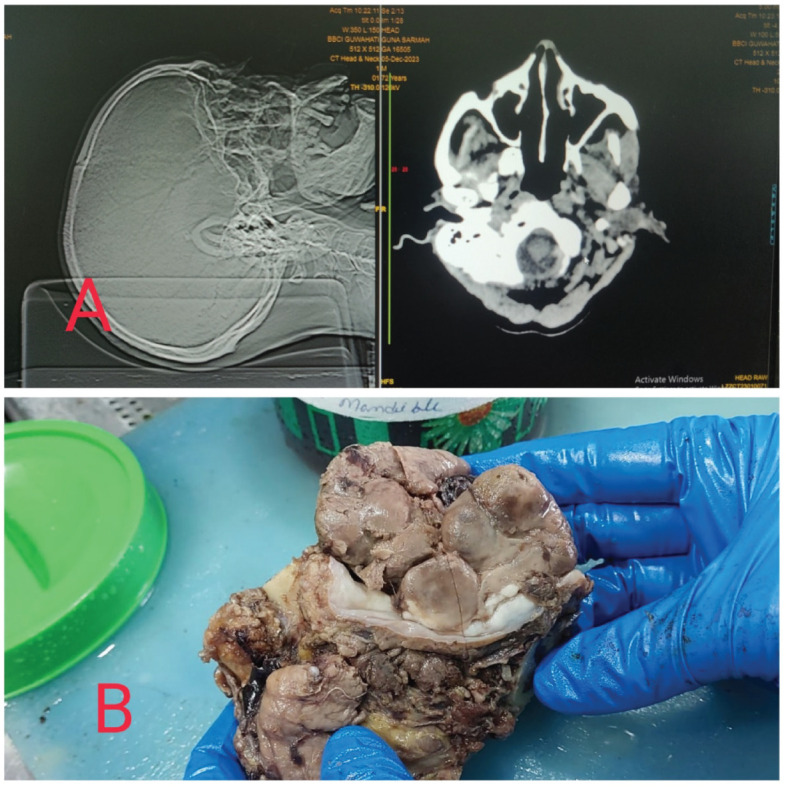
(a): Showing the CT scan of the patient with the mass in the left side of the oral cavity. (b): Gross picture of the post operative specimen of the left buccal mucosal soft, fleshy and lobulated mass.

The specimen was fixed in neutral buffered formalin overnight and grossed the next day. A large lobulated, soft and fleshy tumour was identified in the buccal mucosa and the left lower alveolus measuring 6 × 5 × 4 cm^3^. The thickness of the tumour was 4 cm. The epicenter of the tumour was left buccal mucosa. The skin was not involved by the tumour. All the resected margins seemed to be uninvolved by the tumour grossly. The mandible was seen to be eroded by the tumour (Figure 1).

**Figure 2.**
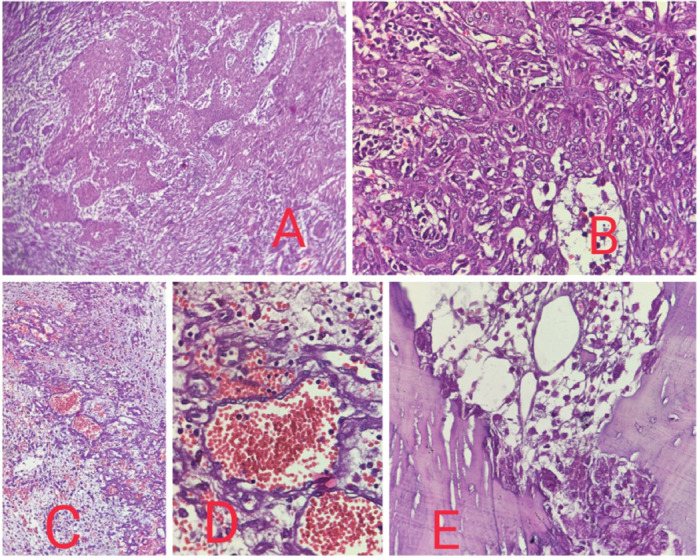
(a): 10× view showing the high grade malignant cells arranged in diffuse sheets, nests and cords. (b): 40× view showing malignant squamous cells diffusely arranged in sheets and nests showing nuclear pleomorphism. (c): 20× view showing high grade malignant cells arranged diffusely with the formation of pseudo vascular and pseudoglandular spaces. Increased vascularity, hemorrhage and inflammation is evident. (d): 40× view showing pseudovascular spaces formation. (e): 40× view showing mandibular bone involvement.

**Figure 3.**
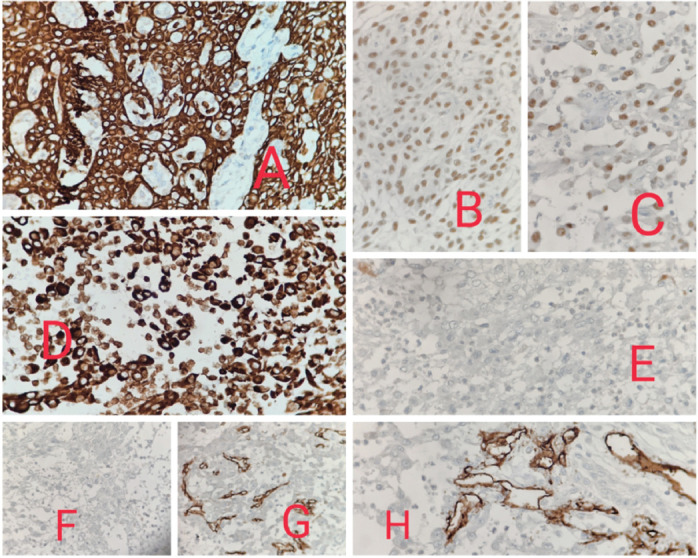
(a): 40× view showing CK strong and diffuse positivity in the tumour cells. (b): 40× view showing P40 positivity. (c): 40× view showing P63 positivity. (d): 40× view showing Vimentin positivity. (e): 40× view showing S100 negativity in the tumour cells. (f): 40× view showing HMB45 negativity in the tumour cells. (g): 40× view showing CD34 negativity in the tumour cells. (h): 40× view showing CD31 negativity in the tumour cells.

Histopathological examination showed malignant epithelial cells having moderate amount of eosinophilic cytoplasm, hyperchromatic nuclei, inconspicuous nucleoli, arranged in nests, trabeculae and irregular anastomosing channels. No definite diagnostic morphology like intercellular bridges was evident. Marked acantholysis and pseudo- vascular spaces lined by atypical cells were seen. Based on morphology, so we made a provisional diagnosis of poorly differentiated malignancy and had three differentials, PSCC, angiosarcoma and high grade sarcoma and accordingly ran a panel of the following immunohistochemistry markers -Cytokeratin, P40, P63 and Vimentin which came out to be positive and S100, HMB45, CD31 and CD34 were negative. So, our final diagnosis was PSCC (Figures 2 and 3).

The worst pattern of invasion was type 4, mitotic count was 2/10 hpf and the depth of invasion was 8 mm. Atypical mitosis was not seen. Lymphovascular and perineural invasion were not seen. All the resected margins were free of tumour microscopically. The underlying mandible was involved by the tumour. Forty-four lymph nodes which were dissected out, were negative for metastasis and showed features of reactive lymphadenitis. The final pathological staging was pT4a pN0.

Though he was managed with the best supportive care, his general condition gradually deteriorated once the diagnosis of PSCC was given and he died within a span of 4 months after the diagnosis, due to complications from pulmonary metastasis.

## Discussion

Conventional-type SCC is by far, the most common malignancy of the head and neck region. Histological variants include: verrucous SCC, spindle cell SCC, papillary SCC, basaloid SCC, adenoid PSCC, lymphoepithelial carcinoma and adenosquamous carcinoma. PSCC is a rare variant of adenoid SCC, characterised by a pseudoglandular pattern and acantholysis. It mimics vascular proliferation and hence also known as pseudovascular adenoid SCC [2]. To the best of our knowledge, only a few cases of PSCC have been reported in literature to date. It mainly affects elderly persons with a male predilection. Clinically, it presents as a rapidly growing and infiltrating mass, as in the present case. On histology, ASCC is classified into 3 categories: (1) the most common subtype consists of acantholytic atypical keratinocytes arranged in solid pattern [6], (2) the pseudoglandular/pseudovascular subtype which is characterised by the presence of vascular spaces containing red cells and (3) the pseudoangiosarcomatous subtype, which shows atypical cells, arranged in vascular structures resembling atypical endothelial cells. Hence, PSCC morphologically is a great mimicker of angiosarcoma. The neoplastic stroma is marked by vascularization and when epidermoid differentiation is absent or minimal or is masked by pseudovascular architecture, the diagnosis becomes even more challenging. Tumour angiogenesis is regulated by a balance between activators and inhibitors. Whereas, pseudoangiovascular changes in a carcinoma is characterised by preservation of vascular like cords and spaces, mimicking glands or vascular differentiation. The presence of atypical cells arranged in vascular structures which often resemble atypical endothelial cells is therefore often confused with angiogenesis as it mimics glands.

Acantholysis is found to be the underlying pathogenetic mechanism, possibly because of the tumour cells changing their expression of adhesion molecules, as studied by Bánkfalvi *et al* [6]. Wijnhoven *et al* [7] studied the correlation of cell to cell adhesion and human cancer and found out that cell-to-cell adhesion is mediated by the Beta catenin-E cadherin complex present on the cell membrane, and this is responsible for forming typical intercellular spaces. This complex gets disturbed in PSCC. The definite diagnosis requires immunohistochemistry. The neoplastic cells express cytokeratins, epithelial membrane antigen, P40 and P63, while vascular markers including CD31 and CD34 are negative [4]. The main differential diagnosis to rule out is angiosarcoma, especially on biopsies. Other diagnosis to consider are: adenosquamous carcinoma and mucoepidermoid carcinoma of minor salivary gland origin, where a glandular pattern is present. Thus, the diagnosis is based on a careful histological and IHC analysis. Given the fact that PSCC have been reported in only a few cases till date, its prognosis remains unclear [8].

According to a literature review by Qi *et al* [1], where they discussed a case report of a 47 years old male patient with penile PASSC and enlarged bilateral inguinal lymph nodes, they found out that the morphology of the tumour has dramatic similarity to angiosarcoma under the microscope along with poor prognosis. Immunohistochemically, most tumour cells were strongly positive for keratin (AE1/AE3) and focally positive for EMA, with the typical squamous cells focally positive for 34betaE12 and Vimentin. The vessels that proliferated in the tumour were highlighted by CD31, CD43 and the tumour cells were negative for HMB45, SMA, Desmin and CEA. HPV DNA was not detected by *in situ* hybridization in the primary and metastatic tumours. Partial penectomy and bilateral inguinal lymphadenectomy were performed, followed by adjuvant pelvic radiotherapy. Two months later, total penectomy was necessitated by penile flap necrosis and local recurrence. Eleven months after the first surgery, the patient died of extensive metastasis to the lungs and pelvis.

According to a literature review by Zidar *et al* [2], two cases of PASCC in the oral cavity were described, which were characterised by acantholysis of the tumour cells with the formation of anastomosing spaces and channels, mimicking an angiosarcoma. Both the tumours contained foci of SCC suggesting the correct diagnosis. Their cases were characterised by loss of IHC expression of E-cadherin, one of the major adhesion molecules of epithelial cells.

Horn *et al* [3] presented a case of a 57-year-old woman with bilateral inguinal metastatic disease at the time of diagnosis, who died 4 months later because of distant metastasis to the lungs. Molecular analysis did not reveal any human papilloma virus infection. Because of the positive P53 immunostaining and the association to lichen sclerosus and high-grade vulval intraepithelial neoplasia, alteration of p53 tumour suppressor gene might be involved in the pathogenesis of vulvar PASCC.

Vidyavathi *et al* [4] reported a case of PASCC of the oral cavity in a 40-year-old man, which mimicked an angiosarcoma initially. IHC analysis led to a conclusive diagnosis of PASCC. On microscopic examination, the tumour was composed of vessel-like anastomosing channels, which were lined by a single layer of atypical epithelioid cells, along with dilated and congested blood vessels. There were no vascular/lymphatic emboli. Seven out of 18 lymph nodes from modified radical neck dissection showed tumour deposits of similar looking cells. A differential diagnosis of SCC and angiosarcoma was considered due to the presence of anastomosing channels of cells. IHC analysis showed strong positivity for cytokeratin in the tumour cells. Vimentin showed focal positivity, but the tumour cells were negative for CD 34. Hence, angiosarcoma was ruled out and a diagnosis of PASCC was made.

Kong *et al* [5] reported two PSCC case; one in a 79-year-old male, bronchoscopy revealed mucosal swelling and hypertrophy and an adrenal mass was found 1 month later; second in a 76-year-old male, computed tomography revealed rib destruction due to a non-calcified soft tissue tumour and, although the tumour resembled an angiosarcoma, endothelial markers were negative and cytokeratin and p63 markers were positive. IHC analysis may be helpful in establishing an accurate diagnosis. PSCC had a progressive course in both patients, who died 3 months postdiagnosis.

Vivek *et al* [8] reported a case of a 51-year-old male with PSCC of the oral cavity with regional lymph node metastasis. However, the patient presented on the 25th postoperative day with gradually progressive localised swelling on the left side of the neck and left cheek. On re-exploration, there was a large hematoma of 500 cm^3^ along with diffuse bleeding from the neck wound, which was drained and the wound closed with suction drainage. Over the next 10 days, the patient developed similar swelling along with skin nodules over the neck. An incisional biopsy of the skin nodule showed the presence of high-grade malignant cells arranged diffusely with the formation of pseudo-vascular and pseudo-glandular spaces. An IHC study was performed to differentiate between PASCC, angiosarcoma and high-grade sarcomas. Cytokeratin, vimentin, p63 and p40 were strongly positive. CD31, CD34, Melan A and Desmin were negative. IHC findings were suggestive of PASCC. The disease progressed over the next 20 days, and the patient succumbed to the disease (Table 1).

In our case, the small biopsies were misdiagnosed as pyogenic granuloma probably due to morphology mimicking a vascular lesion. This points towards the limitation of small biopsies in such cases where the representative area might be scant and that could be a reason for wrong diagnosis. Our patient had past histories of SCC in the right buccal mucosa and pleomorphic spindle cell sarcoma in thigh. The slides of the previous biopsies could not be retrieved as they were almost around 10 years back. So, metastatic carcinoma was not considered and we thought the present case to be a primary lesion. Even the clinicians were considering it to be a new tumour and not a secondary lesion. We chose the panel of immunohistochemical markers keeping in mind our closest differential diagnosis. Positron emission tomography scan which is a valuable investigation especially for metastatic work up was not available at our institute so it was not done .

Our patient, on completion of surgery, was planned for adjuvant radiotherapy. He received five doses of adjuvant radiotherapy, after which he complained of generalised weakness and cough. His counts were increased, and a blood culture showed no growth but a urine culture revealed growth of *Klebsiella pneumoniae*, intermediately sensitive to Fosfomycin. His CT scan revealed multiple bilateral lung metastasis. So radiotherapy was concluded in view of progressive disease and he was for considered for palliative chemotherapy. But the patient did not come for further visits. Soon, we got to know that he expired within a few days. Though he was managed with the best supportive care, his general condition gradually deteriorated once the diagnosis of PSCC was given and he died within a span of four months after the diagnosis, due to complications from pulmonary metastasis.

The recognition of PSCC is important because they are true clinicopathological entities with an important prognostic implication. As they mimic other neoplasms, there may be erroneous treatment. Such a presentation underscores the importance of timely consultation, early diagnosis and prompt treatment. Radical surgical resection is recommended for curative treatment, but supportive evidence is very limited. Some authors believe that PSCC has a more aggressive behaviour than conventional SCC. As the diagnosis is difficult to make due to the morphology of the tumour, so a high index of suspicion about the histology is recommended in a patient who present with early recurrence and rapid progression [5].

## Conclusion

PSCC is associated with a worse prognosis than the conventional SCC. A recurrence should be suspected when the patient presents with a rapidly progressing swelling of the oral cavity, so this rare variant of SCC can be ruled out. Careful attention must be paid to the histological features because they can be confused and mistaken for angiosarcoma. An IHC analysis helps in arriving at an accurate diagnosis.

## Conflicts of interest

There is no conflict of interest.

## Funding

No funding was received.

## Informed consent

Informed consent was taken.

## Author contributions

Dr Rukmini Bezbaruah, Dr Rituparna Dhekial and Dr. Arpan Choudhury contributed to study conception and design.

Material preparation, data collection and analysis were performed by Dr Rukmini Bezbaruah and Dr. Rituparna Dhekial.

The first draft of the manuscript was written by Dr Rituparna Dhekial and all authors commented on the previous versions of the manuscript. All authors read and approved the final manuscript.

## Figures and Tables

**Table 1.** Review of literature of reported cases.

Case	Author	Age/sex	Location	Recurrence	Metastasis	Follow up
1	Qi *et al* [1]	47 years/Male	Penis and inguinal lymph node	Penis	Pelvis, lungs	Died within 11 months
2	Zidar *et al* [2]	52 years/Female 45 years/Male	Oral cavity	N/A	N/A	Died within 16–20 months
3	Horn *et al* [3]	57 years/Female	Vulva	N/A	Lungs	Died within 4 months
4	Vidyavathi *et al* [4]	40 years/Male	Oral cavity	N/A	N/A	N/A
5	Kong *et al* [5]	79 years/Male 76 years/Male	Lung	N/A	Adrenal Ribs	Died within <3 months
6	Vivek *et al* [8]	51 years/Male	Buccal mucosa	Left cheek and left neck	Lungs	Died after 20 days
